# Supplementary material for: Comprehensive Annotation and Functional Exploration of MicroRNAs in Lettuce
Source: Front Plant Sci. 2021 Dec 24;12:781836. doi: 10.3389/fpls.2021.781836 (PMC8739914; doi:10.3389/fpls.2021.781836)
Supplement: Supplementary Data 1 — Results of categories 0–2 from PARE-Seq analysis (including three subfiles:1_1, 1_2, 1_3). [file Data_Sheet_10.ZIP › GSM2230756.plot/Lsa-miR172b_Lsat_1_v5_gn_3_126700.1_453_TPlot.pdf]

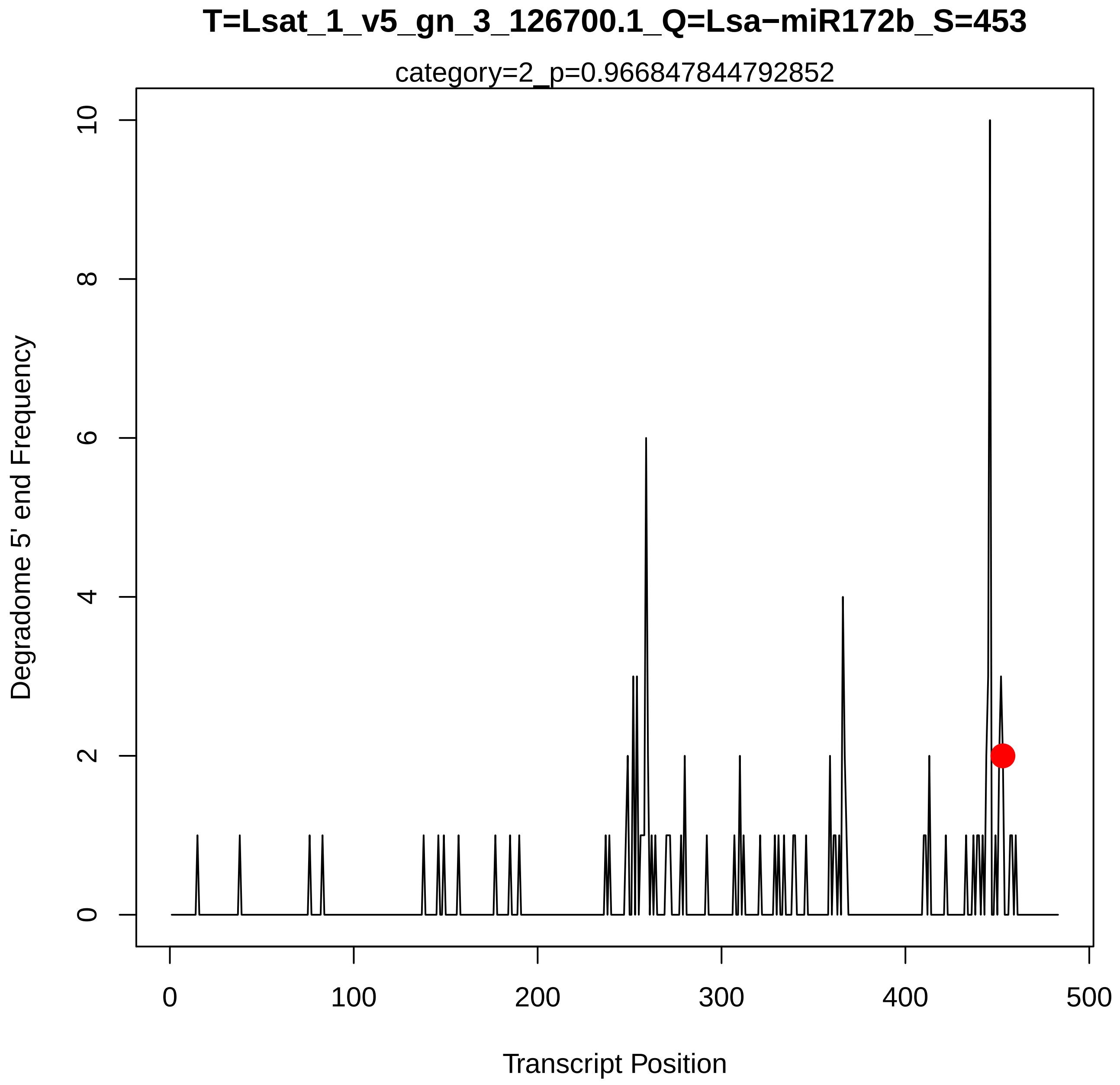

T=Lsat_1_v5_gn_3_126700.1_Q=Lsa−miR172b_S=453
category=2_p=0.966847844792852
Degradome 5' end Frequency
Transcript Position
0
2
4
6
8
10
0
100
200
300
400
500
